# The association between elevated fasting plasma glucose levels and carotid intima-media thickness in non-diabetic adults: a population-based cross-sectional study

**DOI:** 10.18632/oncotarget.22302

**Published:** 2017-11-06

**Authors:** Yalin Guan, Changshen Yu, Min Shi, Jingxian Ni, Yanan Wu, Hongfei Gu, Lingling Bai, Jie Liu, Jun Tu, Jinghua Wang, Xianjia Ning

**Affiliations:** ^1^ Department of Neurology, Tianjin Huanhu Hospital, Tianjin, China; ^2^ Department of Neurology, Tianjin Medical University General Hospital, Tianjin, China; ^3^ Department of Epidemiology, Tianjin Neurological Institute, Tianjin, China; ^4^ Department of Neurology, Tianjin Haibin People’s Hospital, Tianjin, China; ^5^ Tianjin Neurological Institute, Key Laboratory of Post-Neuroinjury Neuro-Repair and Regeneration in Central Nervous System, Ministry of Education and Tianjin City, Tianjin, China; ^6^ Central of Clinical Epidemiology, Tianjin Medical University General Hospital, Tianjin, China

**Keywords:** carotid intima media thickness, fasting plasma glucose, risk factors, ultrasonography, epidemiology

## Abstract

We assessed the association between the mean carotid intima-media thickness (CIMT) and fasting plasma glucose (FPG) levels in a low-income population in rural China. Adults aged ≥45 years without a history of diabetes, stroke, or cardiovascular disease were recruited. All participants were categorized into four groups according to FPG level. A total of 3509 participants were analyzed in this study. In the univariate analysis, sex, age, education level, hypertension, central obesity, current smoking, alcohol consumption, and higher levels of FPG, total cholesterol (TC), triglycerides (TG), high-density lipoprotein cholesterol (HDL-C), and low-density lipoprotein cholesterol (LDL-C) were associated with mean CIMT and frequency of increased CIMT. FPG levels were significantly associated with mean CIMT; each 1-mmol/L increase in FPG resulted in a 2.75-μm increase in mean CIMT when adjusted by age, sex, education level, current smoking status, alcohol consumption, hypertension, and the levels of TC, TG, HDL-C, and LDL-C (P = 0.044). However, the association between FPG and the frequency of increased CIMT disappeared after adjusting by covariates. These findings indicate that FPG is an independent determinant of mean CIMT in a non-diabetic population. Management and control of FPG levels is crucial for preventing atherosclerosis in populations with high stroke risks in China.

## INTRODUCTION

Carotid artery atherosclerosis is associated with a high risk of cardiovascular disease (CVD) and stroke [1-4]. The carotid intima-media thickness (CIMT) is determined using ultrasonography and is thus a noninvasive marker of early atherosclerotic disease [5-7] that has been proven in epidemiological and clinical studies to be a useful predictor of cardiovascular events [8-11]. Furthermore, traditional cardiovascular risk factors have been shown to be determinants of CIMT in the general population [7].

CVD has become the leading cause of death, accounting for almost one-third of all deaths globally in 2013 [12]. Recently, the burden of CVD has become the most important public health issue in China; death due to CVD accounted for more than 40% of total deaths, 44.6% of deaths in rural areas, and 42.5% of deaths in urban areas [13]. Furthermore, as reported in our previous study, there was a dramatically increased incidence of first-ever stroke among a low-income Chinese population in rural China from 1992 to 2012, with an annual percentage change of 6.5% [14]. Concurrently, the prevalence of conventional risk factors has also significantly increased in this population over the past several decades [15, 16].

The relationship between conventional risk factors and CIMT is controversial. Traditional and common vascular risk factors, such as hypertension, diabetes mellitus (DM), dyslipidemia, and smoking, have been reported to be associated with increased CIMT [4, 17-24]. Moreover, systolic blood pressure (SBP), glucose levels, and cholesterol levels have also been reported to be associated to CIMT [25-30]. Several studies reported thicker carotid walls in diabetic patients than in individuals with normal fasting glucose levels [31-34]. In diabetic populations, CIMT was associated with fasting glucose concentrations [34-36]. However, the quantitative relationship between fasting glucose concentration and CIMT in a non-diabetic population, which accounts for more than 80% of the population in China, is unclear, especially in China. We thus aimed to assess the relationship between FPG levels and CIMT among the general non-diabetic population in China.

## RESULTS

### Participant characteristics

A total of 3509 participants were analyzed in this study, including 1461 (41.6%) men and 2048 (58.4%) women. The mean age was 59.82 years overall (60.14 years in men, 58.88 years in women). The average level of education was 5.52 years (6. 40 years in men, 4.88 years in women), with a range of 0 to 16 years. The illiteracy group comprised 17.2% of participants, the 1–6 years of education group comprised 44.5%, the 7–9 years of education group comprised 30.8%, and the >9 years of education group comprised 7.6%. The overall prevalence rates for the evaluated risk factors were as follows: hypertension, 67.1%; central obesity, 34.3%; current smoking, 25.3%; and alcohol consumption, 16.0%. Moreover, there were significant associations between the different mean CIMT and sex, age, education level, hypertension, current smoking, and alcohol consumption. Elevated mean CIMT were associated with the increased levels of systolic blood pressure (SBP), diastolic blood pressure (DBP), and FPG (all P < 0.05; Table 1).

**Table 1:** Description of demographic characteristics for all participants by mean CIMT groups in this study

Risk factors	Overall	< 0.5075	0.5075∼	0.5525∼	≥0.6075	P
Total:	3509	831 (23.7)	893 (25.4)	895 (25.5)	890 (25.4)	<0.001
Men	1461 (41.6)	274 (18.8)	322 (22.0)	380 (26.0)	485 (33.2)	
Women	2048 (58.4)	557 (27.2)	571 (27.9)	515 (25.1)	405 (19.8)	
Age, means (SD), years	59.82 (9.77)	55.65 (8.59)	58.13 (8.96)	61.10 (9.50)	64.13 (9.85)	0.005
Age group, n (%)						<0.001
45∼54 years	1166 (33.2)	427 (51.4)	361 (40.4)	230 (25.7)	148 (12.7)	
55∼64 years	1391 (19.6)	289 (34.8)	351 (39.3)	400 (44.7)	351 (25.2)	
65∼74 years	655 (18.7)	90 (10.8)	134 (15.0)	173 (19.3)	258 (39.4)	
≥75 years	297 (8.5)	25 (3.0)	47 (5.3)	92 (10.3)	133 (14.9)	
Education, means (SD), years	5.52 (3.54)	6.20 (3.32)	5.84 (3.47)	5.18 (3.58)	4.90 (3.63)	0.011
Education, n (%)						<0.001
0 years	602 (17.2)	97 (11.7)	132 (14.8)	179 (20.0)	194 (21.8)	
1∼6 years	1561 (44.5)	333 (40.1)	380 (42.6)	414 (46.3)	434 (48.8)	
7∼9 years	1081 (30.8)	335 (40.3)	306 (34.3)	235 (26.3)	205 (23.0)	
> 9 years	265 (7.6)	66 (7.9)	75 (8.4)	67 (7.5)	57 (6.4)	
Hypertension, n (%)	2355 (67.1)	433 (52.1)	556 (62.3)	654 (73.1)	715 (80.0)	<0.001
Central obesity, n (%)	1204 (34.3)	271 (32.6)	320 (35.8)	345 (38.5)	268 (30.1)	0.467
Current smoking, n (%)	887 (25.3)	171 (20.6)	202 (22.6)	227 (25.4)	287 (32.2)	<0.001
Alcohol consumption, n (%)	560 (16.0)	104 (12.5)	139 (15.6)	141 (15.8)	176 (19.8)	<0.001
SBP, means (SD), mmHg	145.93 (22.08)	138.68 (20.16)	142.46 (20.05)	149.35 (22.40)	152.76 (22.73)	<0.001
DBP, means (SD), mmHg	86.75 (11.41)	85.21 (11.10)	86.12 (10.93)	87.75 (11.63)	87.82 (11.89)	0.032
TC, means (SD), mmol/L	4.85 (1.07)	4.79 (1.07)	4.85 (1.09)	4.84 (1.05)	4.93 (1.08)	0.917
TG, means (SD), mmol/L	1.73 (1.22)	1.76 (1.18)	1.72 (1.08)	1.79 (1.26)	1.66 (1.35)	0.173
HDL-C, means (SD), mmol/L	1.46 (0.46)	1.49 (0.46)	1.46 (0.46)	1.46 (0.48)	1.44 (0.44)	0.202
LDL-C, means (SD), mmol/L	2.68 (1.23)	2.54 (1.19)	2.63 (1.21)	2.64 (1.24)	2.89 (1.24)	0.998
FPG, means (SD), mmol/L	5.70 (1.06)	5.58 (0.97)	5.65 (0.99)	5.69 (0.94)	5.87 (1.28)	<0.001

### Risk factors associated with mean CIMT and increased CIMT in the univariate analysis

Male sex, older age, hypertension, current smoking, alcohol consumption, and higher levels of FPG, TC, and LDL-C were associated with elevated mean CIMT in the univariate analysis (all P < 0.001). However, higher education levels and higher levels of TG and HDL-C were associated with a lower mean CIMT (all P < 0.05; Table 2).

**Table 2:** Associated factors of mean CIMT for all participants in this study by univariate analysis

Risk factors	Means (SD)/ β (SE)^*^	OR 95% CI/95% CI for β	P
Gender:		—	< 0.001
Men	58.28 (9.31)		
Women	55.46 (8.27)		
Smoking, n (%)		—	< 0.001
Yes	58.20 (9.56)		
No	56.10 (8.50)		
Alcohol consumption, n (%)		—	< 0.001
Yes	58.30 (9.70)		
No	56.31 (8.62)		
Hypertension, n (%)		—	< 0.001
Yes	57.89 (9.00)		
No	54.06 (7.88)		
Central obesity, n (%)		—	0.199
Yes	56.37 (8.35)		
No	56.77 (9.07)		
Levels of fasting glucose, n (%)			< 0.001
< 5.1 mmol/L	55.83 (8.63)	1.00	
5.1 mmol/L ∼	55.97 (8.31)	1.01 (0.80, 1.26)	0.960
5.5 mmol/L ∼	56.88 (8.96)	1.19 (0.95, 1.49)	0.125
≥ 6.0 mmol/L	57.79 (9.27)	1.49 (1.20, 1.85)	<0.001
Age	2.62 (0.15)	2.33, 2.90	< 0.001
Education	-3.25 (0.42)	-4.07, -2.44	< 0.001
FBG	7.46 (1.41)	4.69, 10.22	< 0.001
TC	3.88 (1.34)	1.14, 6.62	< 0.001
TG	-2.91 (1.23)	-5.32, -0.50	0.018
HDL-C	-6.49 (3.26)	-12.88, -0.11	0.046
LDL-C	7.46 (1.22)	5.07, 9.85	< 0.001

^*^Categorized variables were presented as means (SD); continuous variables were presented as β (SE).

Similar findings were found for increased CIMT. Male sex, older age, hypertension, central obesity, current smoking, alcohol consumption, and higher levels of FPG, TC, and LDL-C were associated with a greater frequency of increased CIMT in the univariate analysis (all P < 0.05). However, higher education levels and higher levels of TG were associated with a lower frequency of increased CIMT (Table 3).

**Table 3:** Associated factors of increased CIMT for all participants in this study by univariate analysis

Risk factors	References	OR (95% CI)	P
Men	Women	2.02 (1.73, 2.35)	< 0.001
Age	—	1.06 (1.05, 1.07)	< 0.001
Education	—	0.94 (0.92, 0.96)	< 0.001
Current smoking	Never smoking	1.60 (1.36, 1.89)	< 0.001
Alcohol drinking	Never drinking	1.44 (1.13, 1.75)	< 0.001
Hypertension	No	2.38 (1.18, 2.85)	< 0.001
Central obesity	No	0.78 (0.66, 0.91)	0.002
FBG level:	< 5.1mmol/L		
5.1 mmol/L ∼		1.01 (0.80, 1.26)	0.960
5.5 mmol/L ∼		1.19 (0.95, 1.49)	0.125
≥ 6.0 mmol/L		1.49 (1.20, 1.85)	< 0.001
TC	—	1.09 (1.02, 1.17)	0.012
TG	—	0.93 (0.87, 0.99)	0.034
HDL-C	—	0.99 (0.75, 1.05)	0.174
LDL-C	—	1.20 (1.13, 1.27)	< 0.001

### Association between FPG levels and mean CIMT in different multiple linear regression models

**Figure 1:**
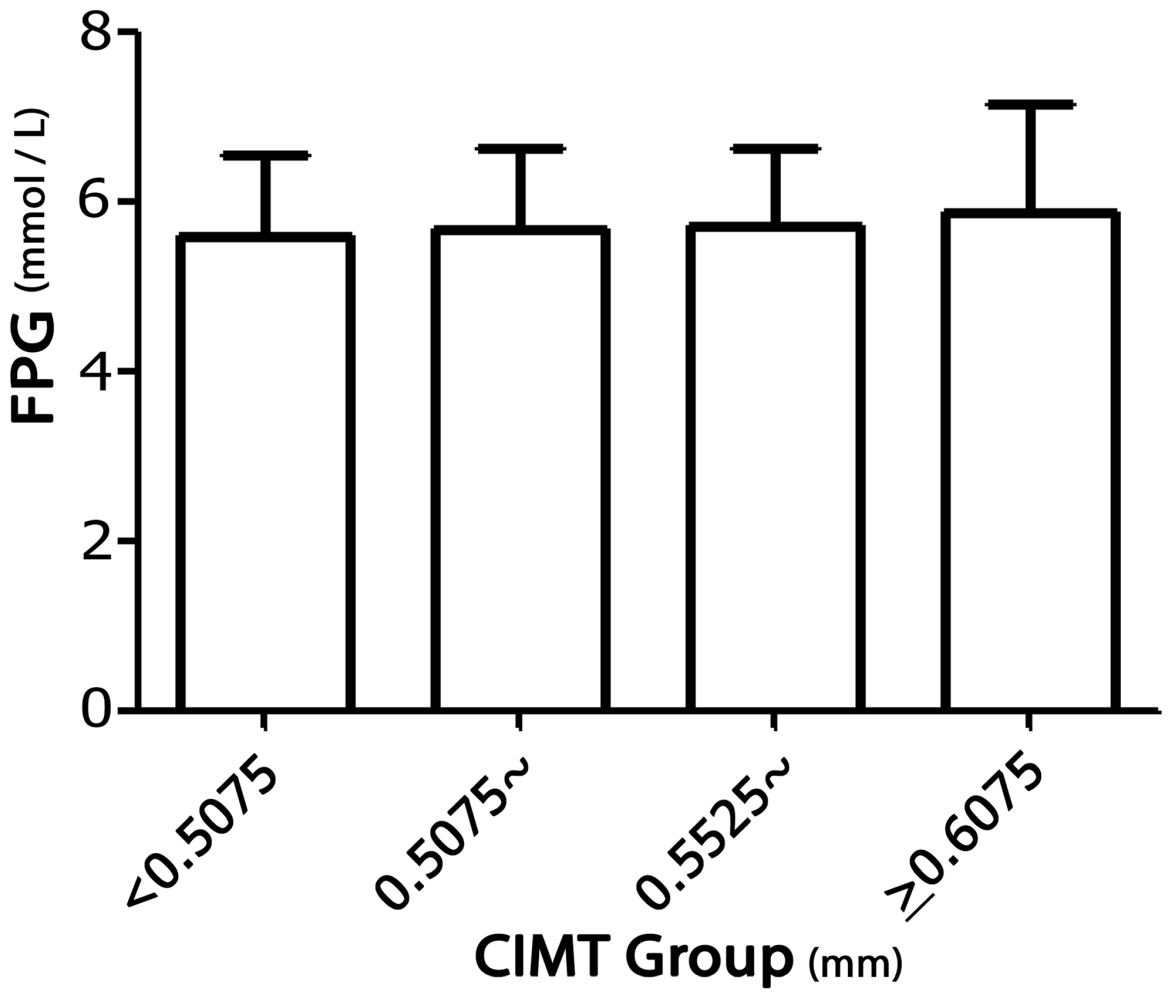
Association between FPG levels and mean CIMT in rural China

FPG levels were significantly associated with mean CIMT (Figure 1); each 1-mmol/L increase in FPG resulted in a 7.46-μm increase in mean CIMT before adjusting for other factors (P < 0.001). There was a 4.58-μm increase in mean CIMT with each 1-mmol/L increase in FPG in model 1 when adjusted by age, sex, and education level (P = 0.001), and there was a 2.98-μm increase in mean CIMT with each 1-mmol/L increase in FPG in model 2, which was adjusted by age, sex, education levels, current smoking status, alcohol consumption, and hypertension (P = 0.028); a 2.75-μm increase in mean CIMT was observed with each 1-mmol/L increase in FPG in model 3 when adjusted by age, sex, education levels, current smoking status, alcohol consumption, hypertension, and levels of TC, TG, HDL-C, and LDL-C (P = 0.044; Table 4).

**Table 4:** Association of the level of FPG with the mean CIMT for all participants in this study

Risk factors	β	SE	95% CI for β	P
Unadjusted	7.46	1.41	4.69, 10.22	< 0.001
Adjusted:				
Model 1	4.58	1.35	1.93, 7.22	0.001
Model 2	2.98	1.35	0.33, 5.62	0.028
Model 3	2.75	1.37	0.07, 5.43	0.044

^*^model 1 was adjusted by age, gender, and education; model 2 was adjusted by age, gender, education, current smoking, alcohol consumption, and hypertension; model 3 was adjusted by age, gender, education, current smoking, alcohol consumption, hypertension, TC, TG, HDL-C, and LDL-C.

### Association between FPG levels and the frequency of increased CIMT in different multiple linear regression models

Table 5 shows that the frequency of increased CIMT was 49% higher in those with FPG levels ≥6.0 mmol/L than in those with FPG levels <5.1 mmol/L in the univariate analysis. However, the association between FPG and increased CIMT leveled off after adjusting by age, sex, education level, current smoking status, alcohol consumption, hypertension, central obesity, and levels of TC, TG, and LDL-C.

**Table 5:** Association of the level of FPG with increased CIMT for all participants in this study

Risk factors	< 5.1	5.1∼		5.5∼		≥6.0	
	OR	Value	P	Value	P	Value	P
Cases, n (%)	179 (22.6)	212 (22.7)	—	230 (25.8)	—	269 (30.3)	—
Unadjusted OR (95%CI)	1.00	1.01 (0.80, 1.26)	0.960	1.19 (0.95, 1.49)	0.125	1.49 (1.20, 1.85)	<0.001
Adjusted OR (95%CI):							
Model 1	1.00	0.98 (0.77, 1.24)	0.847	1.09 (0.86, 1.38)	0.467	1.24 (0.99, 1.56)	0.067
Model 2	1.00	0.94 (0.74, 1.19)	0.609	1.03 (0.81, 1.30)	0.834	1.11 (0.88, 1.40)	0.392
Model 3	1.00	0.98 (0.76, 1.25)	0.845	1.07 (0.83, 1.37)	0.605	1.15 (0.90, 1.47)	0.257

^*^model 1 was adjusted by age, gender, and education; model 2 was adjusted by age, gender, education, current smoking, alcohol consumption, hypertension, and central obesity; model 3 was adjusted by age, gender, education, current smoking, alcohol consumption, hypertension, central obesity, TC, TG, and LDL-C.

## DISCUSSION

In the present study, we assessed the impact of FPG on the mean CIMT and the increased CIMT among middle-aged and elderly non-diabetic adults in a low-income Chinese population. We found that a higher FPG level was associated with an elevated mean CIMT in the multivariate analysis; each 1-mmol/L increase in FPG resulted in a 2.75-μm increase in mean CIMT after adjusting for age, sex, education level, current smoking status, alcohol consumption, hypertension, and the levels of TC, TG, HDL-C, and LDL-C. However, there was not a significant relationship between the level of FPG and the frequency of increased CIMT.

The association between FPG and CIMT has remained controversial, although it has been well established that an elevated FPG level was associated with an increased risk of CVD [37-39].

Previous studies have demonstrated that there was no significant association between FPG and CIMT after adjustment by several covariates, including age, sex, and previous disease history [40-44]. Hyperglycemia was significantly related to CIMT in univariate analyses, but not in the multivariate analysis after adjustment for covariates [45-47]. An association between FPG and CIMT was not found in other studies after adjusting for conventional risk factors [41, 46, 48, 49]. However, the CIMT was significantly greater in those with type 2 diabetes after adjusting for body mass index (BMI), cholesterol, and hypertension [34]. Fasting glucose was not an independent risk factor for CVD when it was added to a prediction model that included sex and CVD risk factors [39]. Furthermore, other studies have indicated that higher HbA1c levels were significantly and independently related to increased CIMT, but impaired fasting glucose (IFG) was not [47, 50]. Nevertheless, a positive association between FPG and CIMT was shown in previous studies [51, 52]. A recent community-based study conducted in Japan reported that IFG was significantly associated with increased CIMT [52]. In the ARIC study, CIMT was significantly greater in patients with type 2 diabetes after adjusting for BMI, cholesterol, and hypertension [53]. Moreover, another study demonstrated that in those with fasting glucose levels <7.0 mmol/L, there was no significant association between fasting glucose and CIMT [47, 50].

Consistent with that study, in this study, we found a significant relationship between FPG and mean CIMT after adjusting for age, sex, educational level, hypertension, current smoking, and levels of TC, TG, HDL-C, and LDL-C. The findings in this study suggest that FPG level is an independent determinant of mean CIMT. The mechanisms between glucose and atherosclerosis may be explained by increased oxidative stress or nonenzymatic glycosylation of proteins and lipids [54].

The disparity between results of the current study and of previous studies with respect to the association between blood glucose level and CIMT may be explained by different populations (i.e., the inclusion of those with normal glucose levels, those with diabetes mellitus, or the entire population), different designs (i.e., hospital-based, community-based, or population-based), and/or different parameters related to blood glucose (FPG, impaired glucose tolerance test, and HbA1c). In the present study, diabetes mellitus was defined with respect to an FPG level >7.0 mmol/L, but not to HbA1c. Moreover, the duration of diabetes mellitus was not available in this study, and we have noted this as a limitation.

Given that the association of the level of FPG with the increased CIMT, there was no statistical significant in this study. The lower mean CIMT in this population may partly explain the negative association between FPG and increased CIMT in this study.

CIMT has been associated with a number of traditional risk factors, including age, sex, hypertension, smoking, lipid profile, and BMI [46]. In agreement with the findings of previous studies, we found a highly significant association between increased mean CIMT and male sex, older age, hypertension, and increased levels of LDL-C among non-diabetic subjects in the present study. Low socio-economic status, defined as low education, low income, or employment in a manual occupation, has been shown to be associated with increased CIMT [55-57]. Consistent with these findings, a negative association between mean CIMT and education level was observed in this population. However, the association between education and CIMT was of marginal significance.

There were several limitations in this study. First, the study population was from a local town in Tianjin, China, so the findings may not be generalizable to the overall Chinese population. Second, the cross-sectional study design may have led to a selection bias and limited the power of causal inference. However, our inclusion of only stroke- and CVD-free participants may have reduced this bias. Third, only fasting glucose levels were measured; oral glucose tolerance tests were not conducted, and HbA1c levels were not measured. This may have resulted in an underestimation of the number of participants with diabetes.

## MATERIALS AND METHODS

### Study population

This was a population-based cross-sectional study conducted from April 2014 to January 2015. The study design has been described previously [58]. In brief, the total population included 14,251 persons from among 18 administrative villages. All residents aged 45 years and over without known diagnoses of CVD and diabetes were recruited to this study.

Among 5380 eligible residents, a total of 4012 individuals participated in the survey. The response rate was 75%. Finally, after excluding 223 residents with a previous history of CVD or stroke and 280 subjects with previous histories of diabetes, 3509 subjects were assessed in this study (Figure 1).

All investigative protocols were approved by the ethics committee of Tianjin Medical University General Hospital; the methods were carried out in accordance with the approved guidelines, and informed consent was obtained from all participants.

### Data collection

We performed an interview and physical examinations according to the prespecified time table. A questionnaire was used to collect all information in this study. Trained epidemiological researchers administered the survey through face-to-face interviews.

Demographic information including sex, date of birth, and education level were obtained from previous records. All participants were categorized into four age groups: 45–54 years, 55–64 years, 65–74 years, and ≥75 years. Education level was categorized into three groups according to educational years: illiteracy (without education), 1–6 years, and >6 years.

### Risk factors and FPG levels

Previous individual medical histories, which included hypertension, diabetes mellitus, stroke, transient ischemic attack, and coronary heart disease, were obtained according to patient self-reporting or from previous records.

Lifestyle characteristics included cigarette smoking and alcohol consumption. Cigarette smoking was defined as smoking more than 1 cigarette per day for at least 1 year, and participants were categorized as never smokers, ever smokers (ceased smoking for at least 6 months), and current smokers. Alcohol consumption was defined as drinking more than 500 grams of alcohol per week for at least 1 year, and participants were categorized into the never alcohol consumption, ever alcohol consumption (temperance for at least 6 months), and current alcohol consumption groups.

Hypertension was defined as a blood pressure level of 140/90 mmHg or higher, or use of antihypertensive medication, given that the subjects were aware of being hypertensive. Self-reported diabetes cases and the date of diagnoses were validated using previous medical records.

Among all subjects without diabetes, FPG measurements were performed during the interview. All participants were categorized into four groups according to the interquartile ranges of FPG, defined as FPG <5.1 mmol/L, 5.1–5.4 mmol/L, 5.5–5.9 mmol/L, and ≥6.0 mmol/L.

### Anthropometric and laboratory measurements

Height, weight, waist circumference, and blood pressure were measured based on standardized protocols. BMI was calculated as weight in kilograms divided by height squared (in square meters). Central obesity was defined as a waist circumference ≥102 cm in men and ≥88 cm in women [59].

Blood specimens were collected after fasting during the periods of study and were separated into 3 tubes: one was kept in the tube with separator gel and EDTA anticoagulant for measurement of glucose, and the other two were kept in the tube with EDTA anticoagulant for measurement of lipids. After blood collection, blood samples were put in a cool bag and then sent to the local central laboratory by a specially-assigned person within 2 hours. The plasma levels of fasting glucose, TC, TG, HDL-C, and LDL-C were measured by means of hexokinase used automatic biomedical analyzer (TBA-2000FR, TOSHIBA, Japan) at the Ji County People’s Hospital.

### Measurement of CIMT

All participants underwent ultrasonographic measurements between April 2014 and July 2014, and all records were analyzed between August 2014 and January 2015. All scans were recorded on Vascular Research Tools 6 (MIA, LLC) for subsequent off-line analysis.

One trained technician blinded to individuals’ previous disease histories performed all ultrasound examinations using B-mode ultrasonography (Terason 3000; Burlington, MA, US) with a 5–12-MHz linear array transducer. The CIMT of the far wall of the distal common carotid artery (CCA) was measured as the distance from the leading side of the first echogenic line (lumen-intima interface) to the leading side of the second line (media-adventitia interface). Extracranial carotid artery trees, which included the CCA, the bifurcation, and the internal and external carotid arteries on both sides, were screened for plaque. Examinations included bilateral observation of the longitudinal and transverse views of the CCA. The trained technician performed the carotid ultrasonography with the participants lying in the supine position with the neck extended in mild lateral rotation.

CIMT at the near and far walls of the common carotid artery were measured on the left and right sides, and 3 values were obtained: the maximum CIMT, minimum CIMT, and average CIMT. Images were obtained and digitally stored according to a standard protocol. The CIMT of the far wall was determined over a length of 10 mm beginning 0–5 mm from the dilatation of the distal CCA. The inter-observer and intra-observer correlation coefficients ranged from 0.88 to 0.94 and from 0.80 to 0.95 for both sides of the CIMT measurement, respectively.

Increased CIMT was defined as the fourth quartile for the mean CIMT, with a mean CIMT of ≥0.6075 cm.

### Statistical analyses

Baseline characteristics of the study population were calculated according to FPG categories. Continuous variables are presented as means with standard deviations and were compared between groups using analyses of variance. Categorical variables are presented as numbers with frequencies, and were compared using chi-squared tests. Linear regression models were used to assess the relationship between FPG level and the mean CIMT. The dependent variable was mean CIMT, and the independent variables were the categorical variables (sex, current smoking, alcohol consumption, and hypertension) and continuous measures (age, education level, and levels of TC, TG, HDL-C, and LDL-C). The linear regression models were established as follows: model 1, adjusted for age, sex, and education; model 2, adjusted for age, sex, education, current smoking, alcohol consumption, hypertension; and model 3, adjusted by age, sex, education, current smoking, alcohol consumption, hypertension, the levels of TC, TG, HDL-C, and LDL-C. The regression coefficient β was interpreted as the increase in CIMT per unit increase of the continuous variable, or the mean CIMT larger in a given category compared to the category of reference. The relationship between the level of FPG and increased CIMT was evaluated by a logistic regression analysis and presented as unadjusted odds ratios (ORs) (95% confidence interval [CI]) for univariate analyses and as adjusted ORs (95% CI) for multivariate analyses. The dependent variables were increased CIMT (yes or no), and the independent variables included categorical variables (sex, current smoking, alcohol consumption, hypertension, and central obesity) as well as continuous measures (age, education level, and levels of TC, TG, and LDL-C). P values < 0.05 in two-tailed tests were considered statistically significant. SPSS for Windows (version 15.0; SPSS Inc., Chicago, IL, USA) was used for analyses.

## CONCLUSIONS

This study assessed the association between mean CIMT and FPG among non-diabetic subjects with a high incidence of stroke in China. We found that a higher level of FPG was associated with an elevated mean CIMT after adjusting for age, sex, education level, current smoking status, alcohol consumption, hypertension, and the levels of TC, TG, HDL-C, and LDL-C. However, the relationship between FPG and the frequency of increased CIMT was not significant after adjusting for covariates. These findings indicate that FPG is an independent determinant of mean CIMT in a non-diabetic population. Management and control of FPG levels is crucial for preventing atherosclerosis in populations with high stroke risks in China.

## NOTICE OF GRANT SUPPORT

This study was funded by Tianjin Medical University General Hospital.

## Abbreviations

CIMT: carotid intima-media thickness
FPG: fasting plasma glucose
CVD: cardiovascular disease
SBP: systolic blood pressure
FPG: fasting blood glucose
TC: total cholesterol
TG: triglycerides
LDL-C: low-density lipoprotein cholesterol
DBP: diastolic blood pressure
HDL-C: high-density lipoprotein cholesterol
BMI: body mass index
